# Effects of gene therapy on cardiovascular symptoms of lysosomal storage diseases

**DOI:** 10.1590/1678-4685-GMB-2018-0100

**Published:** 2019-05-23

**Authors:** Edina Poletto, Gabriela Pasqualim, Roberto Giugliani, Ursula Matte, Guilherme Baldo

**Affiliations:** 1 Gene Therapy Center, Hospital de Clínicas de Porto Alegre, Porto Alegre, RS, Brazil; 2 Postgraduate Program in Genetics and Molecular Biology, Universidade Federal do Rio Grande do Sul, Porto Alegre, RS, Brazil; 3 Medical Genetics Service, Hospital de Clínicas de Porto Alegre, Porto Alegre, RS, Brazil; 4 Department of Genetics, Universidade Federal do Rio Grande do Sul, Porto Alegre, RS, Brazil; 5 Department of Physiology, Universidade Federal do Rio Grande do Sul, Porto Alegre, RS, Brazil

**Keywords:** Lysosomal storage disease, gene therapy, cardiovascular disease, animal models, heart

## Abstract

Lysosomal storage diseases (LSDs) are inherited conditions caused by impaired lysosomal function and consequent substrate storage, leading to a range of clinical manifestations, including cardiovascular disease. This may lead to significant symptoms and even cardiac failure, which is an important cause of death among patients. Currently available treatments do not completely correct cardiac involvement in the LSDs. Gene therapy has been tested as a therapeutic alternative with promising results for the heart disease. In this review, we present the results of different approaches of gene therapy for LSDs, mainly in animal models, and its effects in the heart, focusing on protocols with cardiac functional analysis.

## Introduction

Lysosomal storage diseases (LSDs) are a group of inherited disorders characterized by impairment of lysosomal function due to accumulation of undegraded or partially degraded metabolites. There are more than 50 disorders mainly caused by deficient lysosomal enzymes, but also by decreased function of membrane proteins or non-enzymatic soluble proteins, ultimately resulting in substrate accumulation, reduced lysosomal trafficking and cellular dysfunction (Platt *et al.*, 2012; Boustany, 2013).

Even though LSDs are monogenic, the phenotypes may vary considerably among individuals with the same disease depending on the mutation profile – while some alleles produce proteins with residual activity, others result in complete loss of function. Hence, the clinical spectrum amongst LSDs varies largely accordingly to the residual protein function in addition to the type of metabolite stored (Segatori, 2014).

LSDs are multisystemic and progressive, normally non evident at birth but leading to premature death if not treated (Platt *et al.*, 2012). Common symptoms shared by most of them are organomegaly, cognitive impairment, skeletal defects and coarse facial features, all at variable degrees (Ortolano *et al.*, 2014). Some have cardiovascular involvement, with cardiac failure generally being one of the main causes of death (Braunlin *et al.*, 2011).

In this context, defects in the glycogen, lipid or glycosaminoglycan metabolism lead to disturbed energy production, altered cellular homeostasis and consequent cardiomyopathy (Guertl *et al.*, 2000). Diseases such as glycosphyngolipidoses, mucopolyssacharidoses, and of glycogen storage, normally present significant cardiovascular manifestations, including hypertrophic and dilated cardiomyopathy, coronary artery disease, and valvular disease (Linhart and Elliott, 2007) (Table 1).

**Table 1:** Lysosomal storage diseases with cardiovascular involvement.

Disease	Enzyme	OMIM	Cardiovascular involvement	References
Fabry	α-Galactosidase A	301500	Left ventricular hypertrophy, myocyte hypertrophy and vacuolation, myocardial fibrosis, prominent papillary muscles, valve thickening and insufficiency and arrhythmias	(Linhart and Elliott, 2007)
Galactosialidosis	Cathepsin A	256540	Moderate mitral insufficiency, valve thickening, ventricular hypertrophy, myocardial tissue is thickened and vacuolated	(Senocak *et al.,* 1994) (Bursi *et al.,* 2003)
Gaucher disease	Glucocerebrosidase	2310000	Calcification of cardiac structures, mitral and aortic stenosis, thickened mitral and aortic valves and cardiomegaly	(Guertl *et al.,* 2000)
GM1-gangliosidosis	β-Galactosidase	230500	Cardiomegaly and congestive cardiomyopathy with decreased contractility	(Brunetti-Pierri and Scaglia, 2008) (Morrone *et al.,* 2000) (Guertl *et al.,* 2000)
GM2-gangliosidosis (Sandhoff)	β-Hexosaminidase	268800	Thickening of valves, valvular regurgitation, cardiomyopathy, intimal coronary artery thickening	(Venugopalan and Joshi, 2002) (Guertl *et al.,* 2000)
GSD IIa (Pompe)	α-Glucosidase	232300	Cardiomyopathy, cardiomegaly and heart failure, inrceased aortic stiffness, broad high voltage QRS complexes and short PR interval on ECG.	(Linhart and Elliott, 2007) (Hicks *et al.,* 2011) (Wens *et al.,* 2014)
GSD IIb (Danon)	LAMP-2	309060	Hypertrophic cardiomyopathy, severe conduction abnormalities	(Cheng and Fang, 2012)
MPS I (Hurler, Scheie)	α-Iduronidase	252800	Present in 60-100% cases - valve thickening, mitral and aortic regurgitation and/or stenosis, coronary artery disease, dilation of the ascending aorta and markedly reduced aortic elasticity, systemic hypertension due to arterial narrowing	(Braunlin *et al.,* 2011)
MPS II (Hunter)	Iduronate sulfatase	309900	Present in 60-100% cases - valve thickening, mitral and aortic regurgitation and/or stenosis, coronary artery disease, systemic hypertension due to arterial narrowing, conduction abnormalities and sinus tachycardia	(Braunlin *et al.,* 2011)
MPS IVA (Morquio A)	N-acetylgalactosamine-6 sulfatase	253000	Aortic and mitral valve dysplasia, coronary artery disease, moderate mitral and aortic regurgitation and valve thickening	(Braunlin *et al.,* 2011) (Rigante and Segni, 2002) (Hendriksz *et al.,* 2013)
MPS VI	arylsulfatase B	253200	Valve stenosis and/or insufficiency, cardiomyopathy and fibroelastosis	(Braunlin *et al.,* 2011) (Valayannopoulos *et al.,* 2010)
MPS VII (Sly)	β-glucuronidase	253220	Coronary artery disease, aortic dilation , thickened and stenotic aortic valve leaflets, intimal thickening of the aorta and muscular arteries, left ventricular hypertrophy	(Braunlin *et al.,* 2011) (Gniadek *et al.,* 2015)
Mucolipidosis II/III	N-acetylglucosamine-1-phosphotransferase	607840	Cardiomegaly, mitral and aortic valve thickening	(Cathey *et al.,* 2010)
Niemann Pick type A and B	Acid sphingomyelinase	607608	Mild mitral insufficiency, coronary artery disease (due to atherogenic lipid profile) , cardiomegaly with thickened left ventricular wall	(Senocak *et al.,* 1994) (McGovern *et al.,* 2013) (Guertl *et al.,* 2000)
Sialidosis	Sialidase	256550	Valve disease, ventricular hypertrophy	(Senocak *et al.,* 1994)

Current treatments for LSDs, such as intravenous enzyme replacement therapy (ERT) and hematopoietic stem cell transplantation (HSCT), normally have satisfactory response in some visceral organs such as the liver and spleen, but are insufficient to correct specific tissue manifestations, such as in brain, bones and some cardiovascular symptoms. The central nervous system (CNS) is hard to reach due to the blood brain barrier, which is mostly impermeable to exogenous enzymes and most drug therapies that use conventional administration routes (Parenti *et al.*, 2013). Bones normally respond poorly to treatments due to low vascularization, preventing the therapy to reach the affected area (Clarke and Hollak, 2015). Accordingly, as paradoxical as it may seem, cardiovascular structures as heart valves and aorta are also poorly vascularized, making them less responsive to treatments as well (Ma *et al.*, 2007; Brands *et al.*, 2013).

Most therapeutic approaches for LSDs are based on the event of cross-correction, in which cells can uptake extracellular lysosomal enzymes – administered exogenously or secreted by other cells – via mannose-6-phosphate (M6P) receptor and route them to the lysosomes, where the pH is acid and they can work normally (Sands and Davidson, 2006). In addition, it has been suggested that a recovery of only about 10% of enzyme activity would be enough to prevent or even revert most clinical manifestations (Leinekugel *et al.*, 1992; Sands and Davidson 2006). Hence, modifying few cells would be enough to achieve a satisfying therapy for some LSDs and gene therapy is an approach that meets this idea.

## Gene therapy

The sole purpose of gene therapy is to genetically modify targeted cells (Cotrim and Baum, 2008). Gene therapy is typically performed to correct mutated genes or to add functional copies of a required sequence, although it can also be used to provide new functions to the cells or even to silence overexpressing genes. Its different approaches depend on the pathological condition and targeted tissue and studies have been conducted for genetic, psychiatric, immune and cardiovascular diseases and cancer (Collins and Thrasher, 2015).

For the LSDs, gene therapy seems to have great potential. As opposed to ERT, the promise of gene therapy is that a good gene transfer method could bypass the blood-brain barrier and modify brain cells, which in turn would produce the missing enzyme and possibly brake the CNS manifestations (Zhang *et al.*, 2011).

There are different ways to deliver the gene of interest into cells: using viral or non-viral vectors and also performing gene transfer *in vivo* or *ex vivo*. The simplest way to deliver a gene is cloning it on a plasmid, together with regulatory sequences that ensure its transcription, and then inject it into a cell – this, however, is poorly efficient. Although non-viral vectors are very safe, due to their episomal conformation and very low immunogenicity, their main drawback is the low transfection efficiency. Many strategies have been developed to enhance non-viral gene delivery (Wang *et al.*, 2013; Jayant *et al.*, 2016), as electroporation, gene gun, hydrodynamic injection, nanotechnology-based carriers (Schuh *et al.*, 2016), DNA minicircle (Osborn *et al.*, 2011), and transposons (Aronovich *et al.*, 2009), but all still present low efficiency of transfection and specific procedure limitations.

Viral vectors are recombinant viruses lacking sequences for auto replication, while preserving the ones required to transduce cells, plus the gene of interest. Therefore, they still express surface proteins specific to certain cell types, making the transgene uptake possible through cellular endocytosis, but they fail in replicating and lysing cells (Wang and Gao, 2014). The process of transducing cells is normally highly efficient, making viral vectors the first choice of many studies. Currently, most used are retrovirus (RV), lentivirus (LV) adenovirus (AV), and adeno-associated virus (AAV).

Retroviral and lentiviral vectors are classicaly integrative vectors, i.e., they integrate the transgene into the genome of proliferating cells (and also quiescent cells in the case of lentivirus), which ensures persistent expression and stability. On the other hand, it also brings the risk of insertional mutagenesis by possibly activating oncogenes or disrupting tumor suppressor genes. Integration-deficient LVs are being developed to surpass this limitation, but its efficiency is yet to be determined (Alfranca *et al.*, 2018).

Alternatively, adenoviruses can transduce both quiescent and proliferating cells and do not integrate the sequence into the genome, residing episomally in the cell nucleus. Although this prevents insertional mutagenesis, the expression of the transgene is transient and eventually lost. Another disadvantage of AV is the very strong immunogenicity, leading to possible severe adverse effects in the host (Wold and Toth, 2013).

Adeno-associated viruses have strong tropism for certain cell types due to their capsid serotype, guaranteeing directed treatment to specific tissues (Wu *et al.*, 2006). The apparent lack of immunogenicity and the stable episomal conformation of the DNA, which promotes long-term transgene expression with minimal risk of insertional mutagenesis, are other vantages of this vector. Nevertheless, AAV vectors only enable the insertion of small transgene cassetes, limited to less than 5Kb (Salganik *et al.*, 2015).

Viral vectors are constantly engineered to develop safer versions, as by removing sequences that interact with neighbour genes (as the Long Terminal Repeat, LTR), or preventing the insertional mutagenesis by mutating the integrase or, yet, using gene editing tools to direct the integration site (Wang and Gao, 2014).

### Gene therapy in LSD

There are many studies in the literature about gene therapy in LSDs with different types of vectors, administration protocols and/or auxiliary drugs. Since the cardiovascular system is one of the major systems affected in several LSDs and not responsive to current treatments, it has also been targeted in some gene therapy studies. Therefore, reports that address gene therapy effect on the cardiac system, most still in preclinical stage, are summarized over the next sections.

## Glycogen Storage Diseases (GSD)

### Pompe Disease (GSD Type II a)

Pompe disease, or Glycogen Storage Disease type II a (GSD-IIa), is caused by deficiency of lysosomal acid α-1-4-glucosidase (GAA). In this disorder, glycogen accumulates mainly within the heart and muscles, among other organs. Cardiac disease is characterized by hypertrophic cardiomyopathy, increased aortic stiffness (Wens *et al.*, 2014), broad high voltage QRS complexes, and short PR interval on electrocardiogram (ECG) (Linhart and Elliott, 2007).

Gene therapy for GSD-IIa has been extensively studied, with many different approaches focusing mainly on amelioration of skeletal and cardiac muscles. There is one study describing the use of lentivirus with positive results in the heart, as reduction of glycogen storage (Kyosen *et al.*, 2010), although the majority of published papers describe efforts to improve transduction efficiency and control of immune response using either adenovirus or AAV, as discussed further.

In 1998, a gene therapy protocol "was developed" using transmyocardial injection with adenoviral vector with GAA (AV-GAA) in newborn rats (Pauly *et al.*, 1998). GAA activity was measured in whole heart extracts at day 7, when the animals were euthanized, and resulted in 10-fold the value of the control groups (non-treated or mock treated). According to the authors, no deleterious effects were observed in any groups. Subsequently, analysis of vector sequences in different organs showed that the GAA activity observed in the heart was due to transduced cells in the liver, which would function as a factory of enzyme that could be uptaken by other organs (Pauly *et al.*, 2001). This result inspired other studies that focus on transducing efficiently the liver or a muscle to produce enough enzyme for the whole body.

Similar outcomes using adenovirus were also seen in posterior study using young (Ding *et al.*, 2001) and old (Xu *et al.*, 2005) GAA-knockout (GAA-KO) mice. In spite of the initial promising results, reduced glycogen clearance in muscles of older animals was noticed. Among other possibilities, one speculation is that the fusion of endosomes containing GAA with pre-existing lysosomes full of glycogen may impair the enzyme acitivity as the GAA-KO mice age, resulting in deficient activity of active and correctly processed GAA.

Regarding therapy with AAV vectors, newborn mice receiving the vector with cytomegalovirus promoter (CMV) AAV1-CMV-GAA intravenously resulted in initial supraphysiologic levels of GAA in hearts, with consistent drop in enzyme levels as mice aged (Mah *et al.*, 2005). Posteriorly, heart function was evaluated by electrocardiogram and AAV1-treated animals presented significant prolonged PR interval, with values in between untreated GAA-KO and wild-type mice. Moreover, left ventricular mass was very similar to the wild-type age-matched controls. Although biochemical, histological and functional analysis showed improvement in the cardiac tissue after AAV1 therapy, it only partially corrected the pathology (Mah *et al.*, 2007).

Many AAV vectors, with different promoters and serotypes, proved to be safe and efficient in increasing GAA activity and reducing glycogen deposits in hearts of GAA-KO mice (Table 2), as AAV2 (Fraites *et al.*, 2002), AAV7-MCK (MCK - Muscle Creatine Kinase Promoter) (Sun *et al.*, 2005a), AAV2/8 (Franco *et al.*, 2005; Sun *et al.*, 2005b; Wang *et al.*, 2014), AAV8/DC190 (DC190 - Human Serum Albumin Promoter) (Ziegler *et al.*, 2008) and AAV9-DES (DES - Desmin Promoter) (Falk *et al.*, 2015), this last resulting also in elongation of PR interval, increased ejection fraction and reduction in left ventricular mass. A combination between AV and AAV also showed to be effective in long-term GAA production in heart, even when administered in the gastrocnemius muscle (Sun *et al.*, 2003).

**Table 2:** Effects of gene therapy on cardiovascular system in Glycogen Storage Diseases (Pompe Disease).

	Vector	Administration route	Model and age at administration	Endpoint (time post-injection)	Results in heart		Other remarks	Reference
					Increase in enzyme activity	Substrate reduction		
AV	AV-GAA	transmyocardial	rats, newborn	5-7 days	Yes	Yes	Transduction occured mainly in the liver	(Pauly *et al.,* 1998)
	[E12, polymerase2] AV-GAA	IV, retroorbital sinus	mice, 3 months	2-3 days up to 6 months	Yes	Yes	Treatment was more efficient in the first 50 days post injection	(Ding *et al.,* 2001)
			mice, 3 months	180 days	Yes	Yes	Compared GAA-KO/SCID mice with GAA-KO mice, the first had better results	(Xu *et al.,* 2004)
			mice, 12-14 and 17-19 months	17 days	Yes	Yes	No difference between age groups	(Xu *et al.,* 2005)
	HD-AV	balloon catheter occlusion to liver	Healthy baboon, 6 years	6 months	Yes	No	High levels of protein in the heart, treatment well tolerated	(Rastall *et al.,* 2016)
AV/AAV	hybrid AV-AAV	Intramuscular	Mice, 3 days	24 weeks	Yes	Yes	Transduction of the heart rather than cross-correction from other tissues.	(Sun *et al.,* 2003)
AAV	AAV1-CMV	IV, superficial temporal vein	mice, 1 day	11 months	Yes	Yes		(Mah *et al.,* 2005)
	AAV2*	intramyocardial*	mice, 8 weeks	6 weeks	Yes	Yes		(Fraites *et al.,* 2002)
	AAV2/1-CMV	IV, superficial temporal vein	mice, 1 day	1 year	Yes	Yes	Elongation of PR interval, reduction of left ventricular mass, but mild improvement in correction of cardiac disease	(Mah *et al.,* 2007)
	AAV2/7-MCK*	IV, retroorbital sinus	mice, 12 weeks	24 weeks	Yes	Yes	GAA-KO and GAA-KO/SCID mice	(Sun *et al.,* 2005a)
	AAV2/8	IV, retroorbital sinus	mice, 12 weeks	24 weeks	Yes	Yes	GAA-KO/SCID mice. Restoration of normal myofiber structure	(Sun *et al.,* 2005b)
	AAV2/8-LSP	IV, tail vein	mice, 9–29 weeks	16 weeks	Yes	Yes	Proved to be safe and well-tolerated. Efficacy was higher in males and at later timepoints	(Wang *et al.,* 2014)
	AAV2/8-LSP*	IV, retroorbital sinus	mice, 12 weeks	12 weeks	Yes	Yes		(Franco *et al.,* 2005)
	AAV2/8-MHCK7*	hydrostatic isolated limb perfusion	mice, 3 months	18 weeks	Mild	Mild	Good results in skeletal muscles, but not in the heart	(Sun *et al.,* 2010)
	AAV2/8-LSPhGAA	IV	mice, adult	36 weeks	Yes	Yes	Defined the minimum effective dose; prevented IgG formation due to ERT.	(Han *et al.,* 2017)
	AAV2/9 -MHCK7*	IV, retroorbital sinus	mice, 3 months	18 weeks	Yes	Yes		(Sun *et al.,* 2008)
	AAV2/9-CB	IV	mice, 6 months	12 and 24 weeks	Yes	Yes	Pre-treatment with anti-CD4 mAb enhanced biochemical correction in the heart	(Han *et al.,* 2015)
	AAV2/9-CB	IV, tail vein	mice, 4 months	18 weeks	Yes	Yes	Daily treatment with salmeterol* enhanced biochemical correction observed with AAV treatment	(Han *et al.,* 2016)
	AAV5- or AAV8-DHBV	IV, portal vein	mice, 10 weeks	16 weeks	Yes	Yes	Neonatal pre-treatment with human GAA resulted in greater cardiac correction in mice Ab- for GAA	(Cresawn *et al.,* 2005)
	AAV8-DC190	IV, tail vein	mice, 12 week	6 months	Yes	Yes		(Ziegler *et al.,* 2008)
	AAV9-DES	IV, jugular vein	mice, 3 months	3 months	Yes	Yes	Elongation of the PR interval, increased ejection fraction and reduction in left ventricular mass. In comparison, AVV9 treatment increased more GAA activity in the heart than ERT.	(Falk *et al.,* 2015)
	AAV9-DES*	intrapleural	mice, 3 months	6 months	Yes	Yes	Improved cardiac ejection fraction and stroke volume.	(Falk *et al.,* 2013)
	AAV9-CAG-hGAA	Intrathecal	Mice, 1 month	11 months	Yes	Yes	presented reduced thickness of the left ventricular wall, well arranged myofibrils and correction of vacuolation of cardiac fibers due to glycogen storage	(Hordeaux *et al.,* 2017)
	Co-packaging of AAV9-LSP with AAV9-DES*	IV, tail vein	mice, 4-6 weeks	8 weeks	Yes	Yes	Co-packaged AAV9 attenuated pre-existing humoral and cellular immune responses, enhancing biochemical correction	(Doerfler *et al.,* 2016)
LV	LV-CMV-GAA	IV, superficial temporal vein	mice, 1-2 days	24 weeks	Yes	Yes		(Kyosen *et al.,* 2010)
	*Ex vivo* lentivirus*	IV, retroorbital sinus	mice, 6-8 weeks	17 weeks	NA	No	HSCT using lentivirus modified HSC	(Douillard-Guilloux *et al.,* 2009)
	*Ex vivo* lentivirus*	IV, tail vein	mice, 8-12 weeks	up to 15 months	Yes	Yes	HSCT using lentivirus modified HSC. Decreased relative right and left ventricular mass with restoration of left ventricular wall thickness. Heart rate normalized. Still poor response compared to *in vivo* therapy.	(van Til *et al.,* 2010)

AV: adenovirus; AAV: adeno-associated virus; LV: Lentivirus; IV:intravenous; IM: intramuscular; BMT: bone marrow transplant; HSC: hematopoietic stem cell; HSCT: hematopoietic stem cell transplant; NA: not analysed; *Results showed restricted to the most effective protocol tested.

One of the major drawbacks of viral vector use is the immune response elicited against the vector itself or against the transgene, induced by the viral-mediated expression of GAA which compromises the effectiveness of the therapy, since anti-GAA antibodies probably inhibit cross-correction of peripheral tissues. Xu *et al.* (2004) illustrated this by using GAA-KO/SCID (SCID - Severe Combined Immunodeficiency) mice to analyse the response to AV-GAA therapy in an immunodeficient environment and concluded that the lack of anti-GAA antibodies found in GAA-KO/SCID mice resulted in higher GAA activity and glycogen clearance was maintained longer than previous studies using immunocompetent GAA-KO mice (Xu *et al.*, 2004). To work around this issue, many strategies have been used: pre-treatments with GAA to induce tolerization (Cresawn *et al.*, 2005) or with anti-CD4 to inhibit antibody formation (Han *et al.*, 2015); adaptations in the vector design, using codon-optimized GAA driven by nonviral promoters (Kiang *et al.*, 2006; Doerfler *et al.*, 2016). Interestingly, it has been shown that a pre-treatment with gene therapy using AAV2/8 may prevent IgG antibody formation later with ERT, acting as a pre-conditioning therapy that could enhance the effectiveness of the ERT (Han *et al.*, 2017).

Recently, the use of salmeterol, a β2-receptor agonist, as an adjuvant has been tested, since this drug may enhance the expression of cation-independent mannose-6-phosphate receptor (CI-M6PR) and therefore could improve the response to ERT or gene therapy in heart tissues (Han *et al.*, 2016). The treatment did enhance cardiac response to gene therapy, but further studies should be performed before adding the drug as adjunctive therapy.

Besides the comparison between different vectors or pre-treatment options, alternative administration routes are constantly being analysed as well. Intramyocardial (Pauly *et al.*, 1998; Fraites *et al.*, 2002), intramuscular (Sun *et al.*, 2003, 2010), intrapleural (Falk *et al.*, 2013) and intrathecal (Hordeaux *et al.*, 2017) administrations were already tested and showed transduction of various tissues. Of these, intramuscular administration was seen to be restricted to the injection site (Sun *et al.*, 2003, 2010), while intrathecal injection of AAV9 vector surprisingly resulted in reduction of substrate storage in the heart and consequently reduction of left ventricular wall thickness (Hordeaux *et al.*, 2017). Intrapleural administration of AAV9 also ameliorated cardiac symptoms, with improvement in ejection fraction and stroke volume (Falk *et al.*, 2013).

Contrarily to some other LSDs, hematopoietic stem cell transplant is not an effective approach in GSD-II (Watson *et al.*, 1986) unless hematopoietic stem cells (HSC) are modified *ex vivo* to produce supraphysiologic enzyme levels enough to cross-correct peripheral tissues (van Til *et al.*, 2010). Therefore, this strategy was tested in a couple of studies using lentiviral vectors, in which HSC were harvested from GAA-KO mice donors, modified *in vitro* and transplanted intravenously in GAA-KO mice recipients after sublethal irradiation. The first study (Douillard-Guilloux *et al.*, 2009) showed no significant improvement in the heart of treated animals though the latter (van Til *et al.*, 2010) presented amelioration of echocardiographic findings, with decrease in relative right and left ventricular mass, and reconstitution of GAA activity with robust glycogen reduction in the heart. Even though some improvement was observed, other strategies have proven to be more effective in the context of GSD-II.

Finally, to evaluate safety and efficacy in other animal models, Rastall *et al.* (2016) used baboons, which underwent balloon catheter administration of helper dependent adenovirus (HD-AV) expressing GAA. Both animals treated presented high levels of GAA in the heart, as observed in Western Blot analysis and enzymatic assay.

## Mucopolysaccharidoses (MPS)

### MPS Type I

MPS I (Hurler, Scheie or Hurler-Scheie diseases) is caused by mutations in the *IDUA* gene, resulting in deficiency of α-L-iduronidase (IDUA), an enzyme required in the degradation pathway of glycosaminoglycans (GAGs) heparan and dermatan sulphate. Common cardiovascular manifestations include dilated cardiomyopathy, cardiac valve abnormalities as valve thickening, stenosis and regurgitation, coronary artery disease due to diffuse intimal proliferation from GAG deposition and dilatation of the aorta with reduced aortic elasticity (Braunlin *et al.*, 2011). Moreover, cardiovascular complications, as heart failure, sudden death from arrhythmias and coronary occlusion, are the main cause of mortality (Braunlin *et al.*, 2011).

Numerous studies were conducted using gene therapy to treat murine, canine and feline models of MPS I (Table 3), which also present cardiac disease, although with small differences (Jordan *et al.*, 2005; Braunlin *et al.*, 2006; Sleeper *et al.*, 2008). However, most of these studies focus on the CNS and neurological manifestations, limiting cardiovascular analysis only to IDUA activity and GAG deposition in the heart. Some papers describe the use of lentivirus (Di Domenico *et al.*, 2005; Kobayashi *et al.*, 2005; Ou *et al.*, 2016), adeno-associated virus (Hartung *et al.*, 2004) and non-viral DNA minicircle vectors (Osborn *et al.*, 2011), *Sleeping Beauty* transposon (Aronovich *et al.*, 2009), and microcapsules containing recombinant cells overexpressing IDUA (Baldo *et al.*, 2012; Lizzi Lagranha *et al.*, 2017) to treat MPS I. Results from all of them show increased IDUA activity and reduced GAG storage in heart after the treatment.

**Table 3:** Effects of gene therapy on cardiovascular system in the Mucopolysaccharidoses.

Disease	Vector	Administration route	Model and age at administration	Endpoint (time post-injection)	Results in the heart		Other remarks	Reference
					Increase in enzyme activity	Substrate reduction		
MPS I	non-viral vectors	Hydrodinamic injection, subcutaneous	mice, adult	variable	Yes	Yes	Use of Sleeping Beauty transposon, DNA minicircle and microencapsulated cells	(Aronovich *et al.,* 2009; Lizzi Lagranha *et al.,* 2017; Osborn *et al.,* 2011)
	AAV	IV, temporal vein	mice, 1 day	5 months	Yes	Yes		(Hartung *et al.,* 2004)
		IV, cephalic vein	cats, 3-5 months	6 months	Yes	Yes	Correction of storage lesions in aorta and myocardium, amelioration of aortic valve disease	(Hinderer *et al.,* 2014)
	LV	IV, tail vein	mice, 8-10 weeks	1 month	Yes	Yes	Lentiviral vector elicited low immune response, increasing further at later time points	(Di Domenico *et al.,* 2005)
		IV, temporal vein	mice, 1 day	20 weeks	Yes	Yes	Newborn mice responded better to treatment	(Kobayashi *et al.,* 2005)
	RV	IV, temporal or tail vein	mice, 6 weeks	8 months-old #	Yes	Yes	Reduced GAG in aortic valves and heart, but not in the aorta. Most RV-treated mice had elastic fiber fragmentation and aortic dilatation. Aorta had slight increase in IDUA activity, but not enough to prevent aortic disease. 56% of RV treated mice had aortic insufficiency.	(Ma *et al.,* 2007)
		IV, temporal vein	mice, 6 weeks	8 months-old #	Yes	Yes	Aortas remained dilated, with marked GAG storage, and 75% of treated mice had aortic insufficiency.	(Herati *et al.,* 2008)
		IV, temporal vein	mice, 2-3 days	8 months	Yes	Yes	Prevented aortic dilatation and insuficiency. No significant changes in left ventricular wall thickness, mass index or end-diastolic chamber size. Fractional shortening was significantly greater in high-dose RV mice.	(Liu *et al.,* 2005)
		IV, jugular vein	dogs, 2-3 days	up to 21 months	Yes	Yes	Reduction of aortic diameter, reduced mitral valve thickening and reduced elastic fiber fragmentation of aorta.	(Traas *et al.,* 2007)
	*ex vivo* RV	IV, tail vein	mice, 6-8 weeks	8 months	Low	No	BMT with RV-modified cells. One mice presented restoration of left ventricular function and normalization of myocites storage vacuoles.	(Jordan *et al.,* 2005)
	*ex vivo* LV		mice, 2 months	6 months	Yes	Yes	BMT with LV-modified cells.	(Visigalli *et al.,* 2010)
MPS II	plasmid	electro gene transfer on quadriceps	mice, 12-16 weeks	5 weeks	No	No	Transduction was restricted to injection site, had no effect of the heart	(Friso *et al.,* 2008)
	AAV	IV, tail vein	mice, 2 months	1 and 7 months	Yes	Yes	(Cardone *et al.,* 2006)	
		IV, tail vein	mice, 20 weeks	6 and 24 weeks	Yes	Yes	(Jung *et al.,* 2010)	
	AAV9	Intrathecal	Mice, 2 months	4 months	Yes	Yes	Complete correction of storage lesions in heart, but possibly due to cross-correction from the serum enzyme	(Motas *et al.,* 2016)
		ICV	Mice, 2 months	40 weeks	Yes	Yes	Pilot study compared different routes (intrathecal intravenous and intracerebroventricular).	(Laoharawee *et al.,* 2017)
		ICV	Mice, 2-3 months	3 weeks	NA	Partial		
	*ex vivo* LV	IV	mice, 9 weeks	24 weeks	Yes	Yes	BMT with LV modified cells	(Wakabayashi *et al.,* 2015)
MPS IVA	AAV	IV	mice, NS	12 weeks	Yes	NA		(Tomatsu *et al.,* 2012)
MPS VI	AAV	IV and IM	cats and rats, newborn	6 months (rat) and 1 year (cat)	Yes	Yes	Vector spread to heart after both IM and IV injections for both animal models	(Tessitore *et al.,* 2008)
		IV, temporal or femoral vein	rats 5 and 30 days	6-7 months	Yes	Yes	Pre-treatment with immunosupressionperformed. Heart valve GAG storage was reduced in pre-treated animals.	(Cotugno *et al.,* 2010)
		IV, jugular or cephalic vein	cats, 5 and 50 days	12 months	NA	NA	Reduced or normalized mitral valve thickening independent of age of treatment	(Cotugno *et al.,* 2011)
		IV, retro-orbital	mice, 30 days	6 or 12 months	NA	Yes	Reduced GAG storage in aortic valves and myocardium	(Ferla *et al.,* 2014)
		IV, retro-orbital	Mice, 30 days	6 months	Yes	Yes	Combined low vector dose with monthly ERT infusions	(Alliegro *et al.,* 2016)
		IV	Mice, adult	6 months	NA	Yes	Described safety of the therapy. Minimal GAG reduction in heart valves.	(Ferla *et al.,* 2017)
	RV	IV, jugular vein	cats, newborn	6 months to 8 years	Yes	Yes	Supraphysiologic ARSB levels on the bloodstream, but only 9-85% of normal in heart and aorta of treated cats. Treated cats had significant reduced mitral valve thickening, but still developed aortic dilatation, aortic valve regurgitation and thickened aortic valve leaflets.	(Ponder *et al.,* 2012)
MPS VII	AAV	IV, temporal vein	mice, 2 days	16 weeks	Yes	NA		(Daly *et al.,* 1999)
		Intrahepatic injection	mice, 7-8 weeks	24 weeks	Yes	Yes		(Sferra *et al.,* 2004)
	LV	IV, temporal vein	mice, 2 days	12 or 18 months	Yes	Yes	Used two MPS VII mouse strains.	(Derrick-Roberts *et al.,* 2014)
	LV	IV, tail vein	Mice, 4 months	2 months	Partial	Partial	GAG storage in heart only stabilized but not normalized after treatment.	(Derrick-Roberts *et al.,* 2016)
	RV	IV, tail vein	mice, 5-7 weeks	3 months	Partial	No	Mice were pre-treated with AV-CMV-HGF in the quadriceps. Treatment increased only 5% of GUSB activity in heart	(Gao *et al.,* 2000)
		IV, jugular vein	dogs, 2-3 days	variable, up to 12 months	Yes	NA	Treated dogs had normal valve thickness, no aortic valve insufficiency, mild mitral regurgitation and aortic diameter within normal limits at 8-9 months of age	(Ponder *et al.,* 2002)
		IV, jugular vein	dogs, 2-3 days	24 months	Yes	Yes	Treated dogs had mild mitral regurgitation at 4-5 months of age, which improved over time. At 2 years of age, murmurs were absent and valve thickness was normal. Aortic diameter was within normal limits. Treated dogs had mild improvement in GUSB activity and GAG storage in the aorta.	(Sleeper *et al.,* 2004)
		IV, jugular vein	dogs, 2-3 days	variable, up to 8 years	Yes	NA	Aortic dilatation was delayed in RV treated dogs, but it did occur at late times even with stable serum GUSB activity. They presented reduced elastin fragmentation, reduced expression of MMP-12 and of cathepsins B, D, K and S, compared to the untreated group. RV sequences were not found in the aorta.	(Metcalf *et al.,* 2010)
		IV, jugular vein	dogs, 2-3 days	variable, up to 8 years	Yes	Yes	GAG content in the mitral valve of treated dogs at 8 years post injection was lower than untreated dogs, but still higher than the normal. GUSB activity was 25% of normal in the mitral valves. Treatment reduced total cathepsins activity and increased content of intact collagen.	(Bigg *et al.,* 2013)
		IV, temporal vein	mice, 2-3 days	6 months	Yes	Yes	Aorta GUSB activity in treated animals was 5-fold de value of normal mice and 325-fold de value of the untreated ones. GAG content reduced to 5% of untreated mice, although stil higher than normal. Reduced aortic dilatation but did not prevent it.	(Baldo *et al.,* 2011)

AAV: adeno-associated virus; LV: Lentivirus; RV: retrovirus; IV:intravenous; IM: intramuscular; ICV: intracerebroventricular; BMT: bone marrow transplantation; NA: not analysed; NS: not specified; #Age at analysis

Ma *et al.* (2007) treated six-week-old MPS I mice intravenously with an RV expressing canine IDUA under control of the liver-specific Human α _1_-Antitrypsin Promoter (hAAT-cIDUA-WPRE) and the Woodchuck Hepatitis Virus Post-Transcriptional Regulatory Element (WPRE), resulting in mild improvement in cardiovascular structures in animals expressing stable enzyme activity in the serum. Lysosomal storage was significantly reduced in aortic valves and cardiac parenchyma, but not in the aorta. The aorta of treated mice remained dilated and with elastic fiber fragmentations (Ma *et al.*, 2007). Accordingly, in another study (Herati *et al.*, 2008), even though the enzyme activity was 37% of normal in the aorta of RV treated mice, GAG levels remained high in this tissue, and 75% of treated animals developed aortic insufficiency due to aortic dilatation.

On the other hand, a study using newborn MPS I dogs and RV hAAT-cIDUA-WPRE gene therapy showed great improvement in cardiac disease on the treated group, with reduction of aortic diameter, mitral valve thickening and elastic fiber fragmentation (Traas *et al.*, 2007). The enzyme activity detected in the heart and the aorta was 30% and 20% of normal, respectively, and the lysosomal storage was reduced in both tissues. Furthermore, in MPS I cats (Hinderer *et al.*, 2014), AAV8 vector expressing feline IDUA by a liver-specific promoter was administered. Aorta and myocardium exhibited total correction of storage lesions and aortic valves had near complete resolution in animals with constant enzyme expression. In addition, collagen structures of the fibrosa layer of the valves from treated cats were very similar to the normal group. One cat had a decline in IDUA activity and, consequently, had the worst outcome, but antibodies against IDUA were not found in ELISA tests.

In another study, MPS I mice treated with high-dose RV hAAT-cIDUA-WPRE showed promising outcomes: the echocardiograms in treated mice were completely normal – no significant changes in wall thickness, left ventricular mass index or end-diastolic left ventricular chamber size – and none had aortic dilatation or aortic insufficiency, features found on untreated MPS I mice or treated with lower RV doses (Liu *et al.*, 2005). Additionally, lysosomal storage was absent from most regions in the aorta, mitral valve and myocardium of high dose RV treated animals, while it was present in the untreated group. High dose RV had 9-fold normal IDUA activity in heart and aorta. Hexosaminidase levels – which is a lysosomal enzyme with activity elevated due to MPS I secondary effects – was also normal, as did GAG levels in both organs. Interestingly, low-dose RV treated MPS I mice had 14% of normal IDUA activity in the aorta and it was insufficient to prevent cardiac disease, which may be explained by the unequal distribution of the enzyme throughout the structure (Liu *et al.*, 2005).

Finally, few studies have used HSCT together with gene therapy, modifying the cells *ex vivo* to overexpress IDUA using viral vectors. Initially, in a study using a retroviral vector containing IDUA, only one treated mouse out of ten had mild cardiac improvement (Jordan *et al.*, 2005). On the other hand, Visigalli *et al.* (2010) compared normal and modified HSC from MPS I donor to overexpress IDUA through lentivirus transduction. Plasma IDUA levels were much higher with modified HSC than in other groups and resulted in almost complete absence of lysosomal storage and other pathological conditions, while the transplant with normal HSC only offered mild improvement in comparison. This study highlights the fact that the complete resolution of the cardiac manifestations is very much dependent on the supraphysiologic levels of the enzyme, otherwise the treatment would not be as effective in hard-to-target organs (Visigalli *et al.*, 2010).

### MPS Type II

MPS II (Hunter disease) is an X-linked recessive disease caused by iduronate 2-sulfatase (IDS) deficiency, leading to dermatan and heparan sulfate accumulation. Cardiac involvement is very similar to MPS I, but MPS II patients may also present conduction abnormalities and sinus tachycardia (Braunlin *et al.*, 2011).

Gene therapy research for MPS II started in the decade of 1990 with *in vitro* modification of cells to express IDS based on viral vectors (Braun *et al.*, 1993, 1996 Whitley *et al.*, 1996; Di Francesco *et al.*, 1997; Stroncek *et al.*, 1999) and non-viral approaches in 2002 (Tomanin *et al.*, 2002). Only in 2006 the first paper describing *in vivo* gene therapy of MPS II was published (Cardone *et al.*, 2006), following the creation of the mouse model. The transduction was directed to the liver, using AAV2/8-TBG-IDS vector. In the latter, increased IDS activity was observed in the heart, as was clearance of lysosomal GAG deposition. Other analyses regarding cardiovascular function were not performed. These findings were also seen in a posterior study using a different mouse model and a similar vector (Jung *et al.*, 2010). On the contrary, when a plasmid vector was administered in the quadriceps muscle followed by electro-gene transfer, IDS activity was not detected in visceral organs, including heart, remaining restricted to the injection area (Friso *et al.*, 2008). Three recent studies described CNS-directed administration of AAV9 in MPS II mice, resulting in increased IDS activity in heart and correction of storage lesions (Laoharawee *et al.*, 2017; Motas *et al.*, 2016) or partial reduction of GAG storage (Hinderer *et al.*, 2016). Since the organ did not present sufficient AAV copies, the occurrence of cross-correction of IDS deficiency by uptake of the enzyme from circulation was suggested.

Similar to an MPS I study already discussed (Visigalli *et al.*, 2010), Wakabayashi *et al.* (2015) have shown that *ex vivo* HSC gene therapy using lentiviral vector improves the biochemical abnormalities of MPS II mice, including heart, with increased IDS activity to 3-fold higher than normal and normalized lysosomal GAG content. Unfortunately, echocardiographic analysis was not performed (Wakabayashi *et al.*, 2015).

### MPS Type IV A

MPS IV A (Morquio A disease) is caused by keratan and chondroitin sulfate deposition due to deficiency of the lysosomal enzyme N-acetylgalactosamine-6 sulfatase (GALNS). Clinical findings normally include cardiovascular involvement, with moderate mitral e aortic regurgitation and valve thickening (Hendriksz *et al.*, 2013). Heart failure is the most common cause of death amongst patients (Rigante and Segni 2002).

The first record of gene therapy for MPS IV A dates back to 2001, where patients’ fibroblasts and other lineage cells were transduced *in vitro* by retroviral vector containing the GALNS cDNA and efficiently produced the missing enzyme from 5-fold to 50-fold higher than the baseline enzyme activity of normal non-transduced cells (Toietta *et al.*, 2001). Only one paper described the use of gene therapy *in vivo*, in which MPS IV A mice were treated with AAV vector carrying GALNS cDNA. Twelve weeks after treatment, GALNS activity was about 30% of wild-type in the heart (Tomatsu *et al.*, 2014).

### MPS Type VI

MPS VI (Maroteaux-Lamy syndrome) is an autosomal recessive disease caused by mutations in the *arylsulfatase B* (*ARSB*) gene, resulting in reduced or absent enzyme activity of arylsulfatase B (ARSB), responsible for the breakdown of dermatan sulphate. Cardiac disease is frequent in MPS VI patients and is an important cause of morbidity and mortality (Braunlin *et al.*, 2011). Common features are valve stenosis and/or insufficiency, with mitral valve being affected in 96% of the patients (Valayannopoulos *et al.*, 2010).

Gene therapy for MPS VI is being developed mainly using AAV vectors designed to target the liver, trying to use the organ as a factory that secretes enough enzyme to be taken up by the whole body. Tests were performed in cats and rats, and increased ARSB activity and GAG clearance was observed in heart from both animal models after intravenous or intramuscular AAV treatment (Tessitore *et al.*, 2008). However, the intramuscular treatment showed a positive response in visceral organs, probably due to leakage of vector to other tissues, thus transducing other cell types, rather than a cross-correction effect. In addition, it elicited a humoral immune response in rats, resulting in ARSB levels decay. Thereafter, in another study (Cotugno *et al.*, 2010), the same AAV2/8-TBG-hARSB vector, with the liver-specific promoter TBG (Thyroxine Binding Globulin), was administered intravenously in MPS VI rats, newborn and juvenile, concomitant with immunosuppressive drugs (IS) to minimize the possible drawback caused by the immune response previously reported. GAG storage in heart valves was similar in AVV treated and non-treated MPS VI rats whereas it was reduced in AAV+IS group. Nevertheless, results varied considerably and were not reproducible in the cat model, which can be explained by the mixed genetic background of these animal models and differences in the performance of the essays.

Cotugno *et al.* (2011) used the same AAV2/8-TBG-fARSB vector intravenously in 5 and 50-days-old MPS VI cats (newborn and juvenile groups, respectively). Soon after administration of high vector doses (6 x 10^13^ gc/kg) in 5-days-old kittens, serum ARSB activity levels were 30-fold higher than normal, but shortly dropped to normal range due to intense hepatocyte proliferation, resulting in vector dilution since AAV vector is not integrative. On the other hand, same vector doses administered in juvenile group resulted in stable high ARSB activity and maintenance above or within the normal range throughout the follow-up time, suggesting that late gene therapy with AAV2/8 vector may be beneficial for eventual clinical application, since MPS VI patients are not normally diagnosed at birth. In this study, echocardiographic analysis was performed in 9-12 month-old animals. Untreated cats presented important mitral valve thickening, while treated cats had the condition reduced or normalized (Cotugno *et al.*, 2011).

In mice, AAV2/8-TBG-hARSB was used with intravenous injection in 30 days-old MPS VI mice in comparison to ERT (Ferla *et al.*, 2014). Both treatments showed to be effective in reduction of GAG storage in myocardium and heart valves, although gene therapy provided stable ARSB levels (on average 17% of normal levels) without the peak-and-drop serum kinetics observed with ERT. On the other hand, since high vector doses may compromise liver function, association between gene therapy and ERT was tested using a single IV administration of low dose AAV2/8 (< 2 x 10^12^gc/kg) and monthly enzyme infusions, resulting in increased ARSB activity and GAGs reduction in heart valves and myocardium compared to ERT alone (Alliegro *et al.*, 2016).

Finally, the vector AAV2/8-TBG-hARSB seems safe and effective up to 180 post-administration in MPS VI mice, as observed in a safety study (Ferla *et al.*, 2017), showing the feasibility of a possible translation of the therapy to the clinic.

Retroviral vector was also tested in MPS VI animal models. MPS VI kittens were treated with RV vector hAAT-fARSB-WPRE within 4 days after birth. As a result, GAG deposition reduced drastically in all tissues analysed from treated cats compared to untreated. Conversely, serum ARSB activity in RV group ranged an average of 13-fold compared to homozygous normal cats and 60-fold compared to the untreated MPS VI group. However, the enzyme activity in visceral organs including heart and aorta were 9-85% of the values in unaffected cats, demonstrating that the enzyme is not being taken up to cells efficiently even though there are supraphysiologic levels on the bloodstream. Untreated MPS VI cats eventually developed aortic dilatation at the sinus of Valsalva and at the sinotubular junction, aortic valve regurgitation, thickened aortic valve leaflets and thickening of the mitral valve. These conditions were not present in normal cats and all but thickening of mitral valve were significantly reduced in RV treated animals. Hence, neonatal gene therapy seems to prevent aortic dilatation and aortic valve thickening, although this does not indicate resolution of cardiac disease (Ponder *et al.*, 2012).

### MPS Type VII

Mucopolysaccharidosis type VII, or Sly syndrome, is caused by β-glucoronidase (GUSB) deficiency, resulting in lysosomal build-up of chondroitin, dermatan and heparan sulfate. Cardiac symptoms include coronary artery disease, aortic dilation, thickened and stenotic aortic valve leaflets, intimal thickening of the aorta and left ventricular hypertrophy (Braunlin *et al.*, 2011; Gniadek *et al.*, 2015).

Since the late 1990s, several studies have been published analysing the effect of gene therapy on the cardiovascular system in MPS VII animal models. Daly *et al.* (1999) injected intravenously an AAV vector with human GUSB cDNA in newborn MPS VII mice resulting in stable GUSB expression, higher than normal, for up to 16 weeks in the heart. Contrarily, the RV vector hAAT-GUSB with pre-treatment of AV-CMV-HGF (HGF - Hepatocyte Growth Factor) – which induces transient hepatocyte replication thus allowing the RV vector to transduce the dividing cells – increased GUSB activity in heart only slightly (about 5%) and lysosomal storage did not improve (Gao *et al.*, 2000).

The retroviral vector hAAT-cGUSB-WPRE had better outcomes, as presented in few studies using murine and canine MPS VII animal models. Three newborn MPS VII dogs were treated intravenously (Ponder *et al.*, 2002), none of which presented aortic valve insufficiency or mitral valve thickening at 8-9 months, common features of age-matched MPS VII dogs, and only one had mild mitral regurgitation. Subsequently, Sleeper *et al.*(2004) published a neonatal treatment of dogs with the RV vector hAAT-cGUSB-WPRE, which resulted in improvement in echocardiographic analyses – tricuspid, aortic and pulmonary valves thickness were normal in all treated dogs, none had murmurs and aortic diameter was within normal limits. Two dogs presented insignificant mitral regurgitation at 9 to 11 months; and at 2 years of age, one had minimal mitral regurgitation, but the same happened to 3 out of the 7 normal dogs analysed. GUSB activity in the aorta and myocardium was around 17% and 19% of normal, respectively, and GAG content were reduced to 178% of normal in both aorta and myocardium.

Histologically, the aorta from RV-treated dogs had fusiform myocytes with minimal hypertrophy and vacuolated cytoplasm, in contrast to the rounded, severely hypertrophic and vacuolated muscle cells found in the aortas of untreated dogs. Also, untreated MPS VII dogs had important nodular thickening of the mitral valve, while only mild thickening was seen in the treated group. Although the treatment did not clear GAG storage completely, these results have shown that neonatal intravenous RV gene therapy can ameliorate cardiovascular abnormalities in dogs with MPS VII, with no adverse effects.

Additionally, MPS I and VII dogs were treated with the RV vector hAAT-cIDUA-WPRE and hAAT-cGUSB-WPRE, respectively, at 2 to 3 days after birth (Metcalf *et al.*, 2010). All MPS VII treated dogs had stable high serum GUSB activity throughout the evaluation period, which means up to 8 years for some dogs, albeit GUSB activity in aorta was relatively low, reflecting the poor diffusion of the enzyme in this structure. The aorta appeared normal in a 6 month-old RV-treated MPS VII dog with high enzyme activity in serum, however in an 8 year-old treated dog the aorta was dilated and the aortic valve was thickened, with reduced range of motion. The first dog had 148% of normal enzyme activity and the second had 52%, which may contribute for the difference in the outcome. Summarizing, aortas from treated dogs appeared normal in the first 5 years after gene therapy, statistically better than untreated group, but became dilated thereafter, at 8 years of age. Since GUSB expression remained stable, the treatment with gene therapy delayed the aortic pathology but did not prevent it.

Mitral valve disease progression in MPS VII dogs was also evaluated by the same group (Bigg *et al.*, 2013). The authors suggested that mitral regurgitation occurs due to reduced content of collagen and its abnormal structure in the mitral valve, as evaluated by Masson’s trichrome and Picrosirius-red staining, respectively. Normal and treated dogs had higher collagen content than MPS VII animals, although in the treated group it was slightly less than normal. Regarding collagen structure, RV gene therapy improved the integrity of the fibers to 45% of normal and 5-fold the value in untreated MPS VII dogs at 6 months of age. Biochemical analysis showed almost complete GAGs clearance and reduced protease activity such as cysteine cathepsins in RV treated samples.

In mice, GUSB activity was statistically higher in the hearts of mice treated with hAAT-cGUSB-WPRE than in the untreated group and GAG storage was cleared in heart valves and aorta of RV groups with high GUSB circulating levels (Xu *et al.*, 2002). In another study with the same protocol, GUSB activity in aortas from treated animals was statistically higher, with 5-fold the value of normal mice and 325-fold higher than the untreated group. GAG levels were 111-fold normal in MPS VII mice aortas and significantly reduced to 5% in RV treated mice. Although biochemical parameters were improved, aortic diameter in treated animals was about 155% of normal at 10 months of age, thus aortic disease still developed and progressed, giving another evidence that this gene therapy protocol was not fully effective in aortic abnormalities (Baldo *et al.*, 2011).

Other studies were conducted without performing functional analysis of heart and using different approaches. Serotype 2 AAV vector expressing human GUSB were administered intrahepatically in MPS VII adult mice, resulting in GUSB activity at 15% of normal (Sferra *et al.*, 2004). Besides AAV vector, the lentiviral vector derived from the Human Immunodeficiency Vector pHIV-1EF1α-GUSB (1EF1α - Eukaryotic Translation Elongation Factor 1 α1) was tested, administered intravenously in newborn pups 2 days after birth. Mice from two different strains were used, GUS^tm(L175)Sly^ and GUS^mps/mps^, representing the attenuated and severe form of the disease, respectively. Transduction of heart myocytes were more efficient in GUS^tm(L175)Sly^ animals, as was the GUSB activity, reaching about 60% of normal in the organ. GAG storage reduced, but was still present in animals from both strains (Derrick-Roberts *et al.*, 2014). When treating older mice, results were more modest (Derrick-Roberts *et al.*, 2016).

## Sphingolipidoses

### Fabry Disease

Fabry Disease is an X-linked disorder caused by deficiency of the lysosomal hydrolase α-galactosidase A (α-GalA) with consequent accumulation of globotriaosylceramide (Gb3) within lysosomes. Cardiac symptoms are very common in both men and women with the classic form or the cardiac variant. Moreover, these are main causes of morbidity and mortality. The symptoms include: cardiac hypertrophy associated with depressed contractility and diastolic filling impairment, coronary insufficiency, atrioventricular conduction disturbances, arrhythmias and valvular involvement (Linhart and Elliott, 2007)

The initial reports of gene therapy in Fabry used adenoviral vectors (Ziegler *et al.*, 1999) (Table 4). The AV-CMV-α-GalA vector was administered via tail vein in the mouse model. Enzyme activity and Gb3 levels were normalized in several tissues, including heart. However, expression declined rapidly and there was reaccumulation of substrate after 6 months. Immunosuppression with a monoclonal antibody against CD40 ligand enhanced outcome of vector readministration. The same group also demonstrated that both depletion of Kupfer cells or pre-treatment with gamma globulins could significantly increase AV transduction (Ziegler *et al.*, 2002) and that pulmonary instillation was an effective administration method (Li *et al.*, 2002).

**Table 4:** Effects of gene therapy on cardiovascular system in the Sphingolipidoses.

Disease	Vector	Administration route	Model and age at administration	Endpoint (time post-injection)	Results in heart		Other remarks	Reference
					Increase in enzyme activity	substrate reduction		
Fabry Disease	Non-viral	IV, tail vein	mice, 4 to 6 weeks; injection repeated after 28 and 56 days	Up to 84 days	Yes	Yes	Cationic lipid–pDNA complex. Increase efficiency with dexamethasone treatment and multiple intravenous injections	(Przybylska *et al.,* 2004)
	Non-viral	IV, left renal vein	NS	Up to 4 weeks	Yes	Yes	Naked plasmid	(Nakamura *et al.,* 2008)
	AAV1	IV, tail vein	mice, 3 months	Up to 37 weeks	Yes	Yes	At least 40-fold increase in enzyme levels and Gb3 normalization	(Ogawa *et al.,* 2009)
	AAV1	IV, external jugular vein	mice, 2 days	Up to 25 weeks	Yes	Yes	At least 40-fold increase in enzyme levels and Gb3 normalization	(Ogawa *et al.,* 2009)
	AAV2	IV, portal vein	mice, 10 to 12 weeks	Up to 25 weeks	Yes	Yes		(Jung *et al.,* 2001)
	AAV2	IM, quadriceps	mice, 3 months	Up to 25 weeks	Yes	Yes	Less than 10% enzyme activity in 25 weeks, but normalization of Gb3 and significant decrease of cardiac hypertrophy.	(Takahashi *et al.,* 2002)
	AAV2	IV, tail vein*	mice, 11 to 12 weeks	Up to 24 weeks	Yes	Yes	No Gb3 storage and 3-fold normal level increase in 24 weeks	(Park *et al.,* 2003)
	AAV2	IV, tail vein	mice, 3 months	Up to 12 weeks	Yes	Yes	Reduction of Gb3 to basal levels in 8 weeks	(Ziegler *et al.,* 2004)
	AAV2	IV, tail vein	mice, 18 weeks	Up to 60 weeks	Yes	Yes	Normalization of enzyme up to 48 weeks and clearance of Gb3 up to 60 weeks.	(Choi *et al.,* 2010)
	AAV8	IV, tail vein	mice, 4 months	Up to 12 weeks	Yes	Yes	Normalization of enzyme levels and Gb3 storage from 4-12 weeks	(Ziegler *et al.,* 2007)
	AV	IV, tail vein	mice, 4 to 6 months	Up to 24 weeks	Yes	Yes	Injection of monoclonal antibody against MR1 facilitated readministration	(Ziegler *et al.,* 1999)
		IV, tail vein	mice, 4 months	28 days	Yes	Yes	Pretreatment with gamma globulins enhanced transduction	(Ziegler *et al.,* 2002)
		IN, pulmonary instillation	mice, 4 months	Up to 8 weeks	Yes	Yes	Transduction restricted to lungs.	(Li *et al.,* 2002)
	LV	IV, temporal vein	mice, neonatal	28 weeks	Yes	Yes		(Yoshimitsu *et al.,* 2004)
		Intraventricular injection	NS	Up to 52 weeks	Yes	Yes	~20% normal levels in 7 days No enzyme activity 30 days or 1 year post injection	(Yoshimitsu *et al.,* 2006)
		HSCT *	mice, 8 weeks	24 weeks	Yes	Yes	After a secondary HSCT mice also presented therapeutic levels of enzyme in heart.	(Yoshimitsu *et al.,* 2007)
	RV	IV, temporal vein	mice, neonatal	26 weeks	Yes	Yes		(Higuchi *et al.,* 2010)
		HSCT	NS	12 and 26 weeks	Yes	Yes	Mice that received a secondary transplantation still exhibit improvement in heart tissue.	(Takenaka *et al.,* 2000)
		HSCT, tail vein	mice, 6-10 weeks	Up to 6 months	Yes	NA	Transductions were performed once a day for 5 days and transplanted in lethally irradiated mice. Enrichment of CD25+ cells enhanced enzyme activity.	(Qin *et al.,* 2001)
		HSCT, tail vein	mice, 7-10 weeks	Up to 26 weeks	Yes	NA	Transductions were performed twice a day for 3 consecutive days and cells CD25+ were enriched. Lethally irradiated mice had the highest activity.	(Liang *et al.,* 2007)
Galactosialidosis	AAV	IV, tail vein	mice, 30 days	Up to 16 weeks	NA	NA	AAV2/8. Complete resolution of swollen lysosomes in the heart	(Hu *et al.,* 2012)
	RV	IV, tail vein	mice, 3 to 6 weeks	Up to 10 months	Yes	Yes	BMT using modified BM cells.Increased cathepsin A activity detected in heart 10 months after treatment, but it decreases as the mice age	(Leimig *et al.,* 2002)
Gaucher Disease	LV	IV, portal and tail vein	mice, 7 weeks	Up to 16 weeks	Yes	NA	Increased glucocerebrosidase activity in both administration routes	(Kim *et al.,* 2004)
GM1 Gangliosidosis	AAV	IV, tail vein	mice, 6 weeks	variable	Yes	NA	AAV9. Treated animals presented increased lifespan	(Weismann *et al.,* 2015)
	AV	IV, superficial temporal vein	mice, 24h-48h	30 and 60 days	Yes	NA		(Takaura *et al.,* 2003)
Niemann-Pick Disease types A and B	*Ex vivo* RV	IV	mice, 2 days	5 months	No	No	BMT using modified BM cells. No detectable ASM activity or sphingomyelin reduction in the heart of treated animals	(Miranda *et al.,* 2000)
	*Ex vivo* RV	IV, superficial temporal vein	mice, 3 days	16 and 24 weeks	Yes	NA	BM and MSC modified *in vitro* with RV. After 30 days of BM injection, MSC was administered intracerebral. ASM activity in the heart increased slightly, but was only 2% of normal after 24 weeks	(Jin and Schuchman, 2003)
Sialidosis	AAV	IV, tail vein	mice, 16-month	4 weeks	Yes	NA	AAV2/8. Increased NEU1 and PPCA activity in the heart	(Bonten *et al.,* 2013)

AV: adenovirus; AAV: adeno-associated virus; LV: Lentivirus; RV: retrovirus; IV: intravenous injection; IN: intranasal; BM: bone marrow; BMT: bone marrow transplant; NS: not specified. NA: not analyzed; *Results showed restricted to the most effective protocol tested

Adeno-associated vectors were the most frequently used vector in reports that evaluated cardiac tissue. Jung *et al.* (2001) detected increased enzyme activity and reduced substrate accumulation in the heart of Fabry mice 25 weeks after injection of an AAV2 vector. Administration of another AAV2 vector in the quadriceps increased enzymatic activity for 30 weeks without development of antibodies (Takahashi *et al.*, 2002). Echocardiography showed that cardiac hypertrophy was significantly reduced. Although the authors detected clearance of cardiac Gb3, wall thickness was not normalized. Since animals were 3-month-old when injected, it is possible that irreversible structural changes were already established and the treatment cannot revert them. This type of vector proved to be very effective, since other authors also reported increased enzymatic activity to at least normal levels and/or complete clearance of Gb3 deposits for up to 60 weeks with the use of intravenously injected AAV1(Ogawa *et al.*, 2009), AAV2 (Park *et al.*, 2003; Ziegler *et al.*, 2004; Choi *et al.*, 2010), or AAV8 (Ziegler *et al.*, 2007) vectors.

Lentiviral vectors were injected in the temporal vein of neonatal Fabry mice (Yoshimitsu *et al.*, 2004; Higuchi *et al.*, 2010). After 26 to 28 weeks, both studies reported reduction in Gb3 and increase α-GalA activity in the heart. The latter study further demonstrated that fusion of α-GalA with the protein transduction domain (PTD) from HIV transactivator of transcription (Tat) protein could enhance the Gb3 reduction. Direct heart intraventricular injection of LV was also shown to reduce disease burden in the cardiac tissue for short-term period (Yoshimitsu *et al.*, 2006).

The combined use of gene therapy and HSCT was also evaluated. Takenaka *et al.* (2000) used α-Gal-deficient HSC from 10-week-old donor mice that were transduced four times with a retrovirus encoding α-GalA. Subsequently, cells were transplanted into sublethally and lethally irradiated α-GalA-deficient mice. After 26 weeks, increased α-GalA activity and decreased Gb3 storage were observed in the heart and other organs, particularly in the group lethally irradiated. Although in a smaller scale, these results were observed even after a secondary transplant was performed (Takenaka *et al.*, 2000). Qin *et al.* (2001) used a bicistronic retroviral vector that expressed α-GalA and a selectable marker (CD25 – Interleukin 2 Receptor α). They harvested HSC from 6- to 10-week-old male Fabry mice and transduced once a day for 5 days. Cells were then sorted by flow cytometry and immunoaffinity enrichment and were injected via tail veins into several groups, including lethally irradiated Fabry mice. Tissue analysis after 6 months indicated near normal α-GalA levels in the heart of these animals. Moreover, a second transplantation of cells collected from these primary transplanted mice was performed. From the 5 transplanted mice, 3 showed enzyme levels equal or higher to normal in plasma. No evaluation of GB3 was performed (Qin *et al.*, 2001). Later, the effects of different Reduced-intensity Conditioning Regimens, in addition to pre-selection, were also analysed. Liang *et al.* (2007) described that both limb irradiation with lethal dose of X-ray and treatment with Fludarabine and Cyclophosphamide increased enzymatic activity after transduction with the same RV as the previous study. However, it was a modest increase. Only mice lethally irradiated presented near normal levels of enzyme activity in the heart with clearance of Gb3. In the same year, another group used a LV to transduce bone marrow mononuclear cells and transplant into Fabry mice. Treatment was effective and resulted in supraphysiological levels of enzyme and total clearance of substrate in the heart and other tissues. After a secondary transplant, enzymatic levels were still elevated in several tissues when compared to untreated mice (Yoshimitsu *et al.*, 2007).

Finally, the use of non-viral vectors as naked plasmid and cationic lipid–pDNA complex was also reported (Przybylska *et al.*, 2004; Nakamura *et al.*, 2008). Although these are safer options, these vectors were not as effective as viral vectors and were unable to normalize the disease in the mouse model.

### Galactosialidosis

Galactosialidosis (GS) is an autosomal recessive disease caused by mutations in the Cathepsin A gene (*CTSA)* that encodes the protective protein/cathepsin A (PPCA). Defective PPCA leads to secondary combined deficiency of β-galactosidase and neuraminidase, resulting in sialyloligosaccharides and glycopeptides accumulation. Patients present typical lysosomal disorder manifestations, such as coarse facies and skeletal deformities (Okamura-Oho *et al.*, 1994). Myocardial tissue is thickened and vacuolated and cardiovascular disease may occur, with mitral and aortic valve thickening leading to valve insufficiency (Bursi *et al.*, 2003) and left ventricular dilatation (Okamura-Oho *et al.*, 1994).

Initially, gene therapy studies focused on developing overexpressing PCAA transgenic mice to use as HSC donors to transplant PCAA-KO mice, which resulted in mild improvement in phenotype (Hahn *et al.*, 1998; Zhou *et al.*, 1995). Later on, an *ex vivo* gene therapy protocol was tested using RV MSCV-PPCA (MSCV - Murine Stem Cell Virus) modified PCAA-KO hematopoietic progenitor cells to transplant PCAA-KO mice. Although the number of PPCA expressing cells varied between treated mice, systemic correction was observed in all MSCV-PPCA transplanted animals. In heart homogenate, cathepsin A activity was detected at higher levels than in untreated group for as long as 10 months (Leimig *et al.*, 2002).

The latest published study using gene therapy was using the vector AAV2/8-LP1-PPCA (LP1 - Liver Specific Promoter) to treat a cohort of sixty 30-day-old PCAA-KO mice, intravenously and with three vector doses. In addition to improved overall appearance and rescue of fertility, histological heart sections showed complete resolution of swollen lysosomes, as seen in other tissues (Hu *et al.*, 2012).

### Gaucher Disease

Gaucher disease (GD) is the most common LSD, with an estimated worldwide incidence of 1:75.000 (Huang *et al.*, 2015). It is caused by deficiency of the enzyme glucocerebrosidase (GCase) and consequent deposition of the substrate glucocerebroside in liver, spleen and bone. There are three classical forms depending on the severity and onset of symptoms – type I (most common, without CNS involvement), type II (acute neuronopathic form) and type III (chronic neuronopathic form). The latter is characterized by a more attenuated phenotype, with mild neuronopathic features and visceral manifestations, including cardiac symptoms.

Patients with Gaucher disease type III (GD III) generally present widespread calcification of cardiovascular structures, such as ascending aorta, coronary and carotid arteries, and cardiac valves, leading to valvular stenosis, dilated cardiomyopathy and possibly congestive heart failure (Guertl *et al.*, 2000).

Most gene therapy studies for GD were developed in the 1990’s, focusing mainly on protocols of *in vitro* HSC transduction for HSCT. After creation of GD murine models, some studies tried *ex* or *in vivo* gene therapy in mice, one of them resulting in increased GCase in the heart (Kim *et al.*, 2004). However, gene therapy for GD was not as successful as it seemed for other LSDs, probably because GCase is not normally secreted unless cells are expressing high levels of the enzyme, requiring very efficient vectors to deliver and induce significant gene expression (Marshall *et al.*, 2002).

### GM1 Gangliosidosis

GM1 gangliosidosis is characterized by accumulation of GM1 gangliosides and related glycoconjugates in lysosomes due to β-galactosidase (β-gal) deficiency. Besides manifestations shared by most LSDs, one third of patients with GM1 gangliosidosis present congestive cardiomyopathy, regardless the classification of the disease (infantile, juvenile and adult) (Guertl *et al.*, 2000; Morrone *et al.*, 2000; Brunetti-Pierri and Scaglia, 2008).

Since cardiac involvement is not experienced by all patients, and neurological involvement is very pronounced, gene therapy protocols developed so far did not focus on heart manifestations but on the CNS. A couple of studies designed to target the CNS described an increase of β-gal activity in heart and other visceral tissues, using either AV vector (Takaura *et al.*, 2003) or AAV (Weismann *et al.*, 2015) when treating GM1 gangliosidosis mice.

### Niemann-Pick Disease types A and B

Niemann-Pick disease (NPD) is caused by storage of sphingomyelin within lysosomes mainly of monocytes and macrophages. Niemann-Pick types A and B are associated with mutations in the Sphingomyelin Phosphodiesterase 1 gene (*SMPD1)* and type C is caused by mutations in the Intracellular Cholesterol Transporter genes *(NPC1* or *NPC2)*, thus presenting different clinical features. Both types A and B are a result of acid sphingomyelinase (ASM) deficiency, though NPD-A has neuronal involvement ,while NPD-B is only visceral (Schuchman and Wasserstein, 2015).

Early cardiovascular disease may occur in some patients with NPD-A and NPD-B, presenting valvar or coronary artery disease, which in turn is probably caused by the abnormal atherogenic lipid profile found in most patients. The mechanisms regarding valvar disease are still unknown (McGovern *et al.*, 2013).

The first paper describing gene therapy for NPD used 2-days-old ASM-KO mice injected with bone marrow cells previously modified with retroviral vector containing ASM (acid sphingomyelinase). ASM levels and sphingomyelin storage in the heart of treated animals were not statistically different than those found in the untreated mice. In other organs, the results were slightly better, suggesting improvement of disease manifestations. However, all transplanted mice eventually developed ataxia and died earlier than normal mice, which highlighted the need of further studies (Miranda *et al.*, 2000). Subsequently, a similar approach using two transplants – one with modified bone marrow cells intravenously at day 3 after birth and another with modified mesenchymal stem cells intracerebrally at day 30 – achieved improvement of neurological features, and ASM activity initially increased in the heart, though it decreased to only 2% of normal after 24 weeks (Jin and Schuchman, 2003). To our knowledge, none of published studies evaluated the efficiency of gene therapy on cardiac disease of NPD.

### Sialidosis

Sialidosis is caused by progressive accumulation of sialylated glycopeptides and oligosaccharides due to neuraminidase 1 (NEU1) deficiency, also known as sialidase. General clinical manifestations include visceromegaly, coarse facies, dysostosis multiplex and mental retardation. Patients may also present cardiac anomalies, including valve disease and increased ventricular wall thickness (Senocak *et al.*, 1994). Currently, no treatment is available.

Genetic transference of NEU cDNA to patients’ fibroblasts has been tested to induce transient expression of the missing enzyme. NEU levels in fibroblasts were restored to normal range and increased further 10-fold when co-transfection with cathepsin A cDNA (PPCA, a chaperone required for lysosomal routing) was performed (Igdoura *et al.*, 1998); abnormal sialylglycoprotein deposits were reduced to normal levels as well (Oheda *et al.*, 2006). *In vivo* gene therapy using AAV2/8 vector to deliver the PPCA cDNA resulted in indirect increase in NEU activity from its residual levels, including in the heart. Further studies should be performed in order to better understand the disease mechanisms and more efficient approaches of therapy.

## Conclusion and final considerations

Gene therapy is being tested along the last three decades for many diseases, and it seems very promising to treat LSDs. Since cardiovascular disease is an important feature of many of them – normally being one of the main causes of mortality among patients – it is important to evaluate how efficient this therapy is on the cardiovascular system and how it can impact on the patient’s life.

Many gene therapy protocols have already been tested and, so far, they showed better results when performed in young and in immunodeficient/pre-conditioned animals. Intravenous injection is the most used route of administration because it is the one that distributes the vector more homogenously. Retrovirus and adeno-associated virus are the most used vectors and have not yet produced serious adverse effects in these animal models, being efficient and apparently safe.

**Figure 1:**
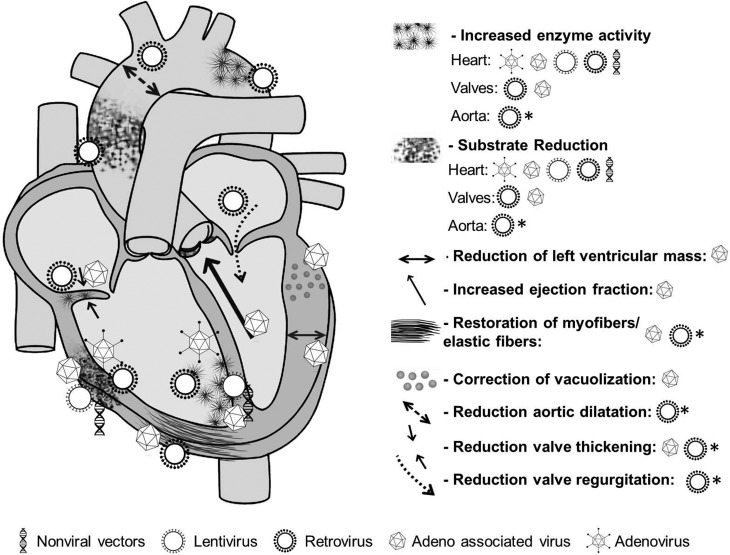
Cardiovascular response to gene therapy depending on vector used, according to studies for lysosomal storage diseases. Schematic representation of the heart and the aorta showing the most prominent cardiovascular manifestations of lysosomal storage diseases (right) with the results obtained from *in vivo* gene therapy using different vectors (bottom). Retroviral and adeno-associated viral vectors resulted in better outcome for many aspects of the disease (although most studies using other vectors did not analyse thoroughly the effect of gene therapy on cardiovascular manifestations and there is no data available). Valves and aorta are most difficult to treat, as most vectors do not reach these structures as easily as the myocardium. Some features were only restored or prevented when treated in the first days of life (represented by * next to the vector symbol).

The heart seems to be much beneficiated from most of gene therapy protocols, ameliorating or normalizing many of analysed parameters (Figure 1), such as wall thickness, electric conductance and heart rate. On the other hand, heart valves and aorta do not respond so well, probably due to poor and/or uneven vascularization, even when there are supraphysiologic levels of missing enzyme in the bloodstream. Aortic dilatation, valve thickening, valve regurgitation and valve insufficiency were delayed in most treated animals with MPS, but they still developed these conditions eventually. For Fabry and Pompe diseases, a similar outcome was observed – all treated mice had improvement in cardiac tissues, but the overall pathology was only partially corrected. For other diseases cited in this revierw, reduction of substrate and increase in enzyme activity was achieved after gene therapy, but this does not indicate cardiac disease resolution due to lack of more specific functional analysis.

## Future directions

There are numerous studies using gene therapy so far, and this approach seems very promising, although more tests should still be performed, perhaps with combined treatments. The use of adjuvant drugs to modulate immune response or to increase enzyme uptake could be helpful, since only increasing serum levels does not seem suficient to have a complete correction of the phenotype.

Nanotechnology-based carriers designed specifically to target valve and aortic tissues, for example, could be a great adjuvant to current limited vectors. Possibly there will not be one perfect vector to fit all demands in an organism affected by a multisystemic disease, but an association of strategic vectors and adjuvant drugs could be the final solution.

Nonetheless, even if not completely corrective yet, stabilizing the disease or delaying the progression of important cardiovascular pathologies could make a big difference for the patient and thus should be continuously pursued.
